# Study the Behavior of Drug Structures via Chemical Invariants Using TOPSIS and SAW

**DOI:** 10.1155/2023/4262299

**Published:** 2023-01-24

**Authors:** Salma Kanwal, Yasmeen Farooq, Muhammad Kamran Siddiqui, Nazeran Idrees, Asima Razzaque, Fikre Bogale Petros

**Affiliations:** ^1^Department of Mathematics, Lahore College for Women University, Lahore, Pakistan; ^2^Department of Mathematics, COMSATS University Islamabad, Lahore Campus, Pakistan; ^3^Department of Mathematics, Government College University, Faisalabad, Pakistan; ^4^Basic Science Department, Preparatory Year Deanship, King Faisal University, Al Ahsa, Saudi Arabia; ^5^Department of Mathematics, Addis Ababa University, Addis Ababa, Ethiopia

## Abstract

Every year, various experiments emerge in which a strong link between topological chemical structures and their properties is found. These properties are numerous such as melting point, boiling point, and drug toxicity. Topological index is the functional tool to determine these properties. This research paper will analyze some of the molecular drug structures, i.e., hyaluronic acid-paclitaxel conjugates *G*_*n*_, anticancer drug SP[*n*], polyomino chain of *n*-cycle *Z*_*n*_, triangular benzenoid *T*_*n*_, and circumcoronene benzenoid series *H*_*k*_ using multicriteria decision-making techniques including TOPSIS and SAW. The topological indices used in this research paper include the Randić index for *α* = 1, −1, 1/2, the augmented Zagreb index and the forgotten topological index.

## 1. Introduction

The introduction of mathematical “graph theory” to chemistry [1] has been playing a significant role. Chemical graph theory is a subset of graph theory that connects to chemical compounds and processes. Chemical graph theory depicts molecular structures as chemical graphs, with nodes and edges representing atoms and bonds. In cheminformatics, they depict chemical structures. The cornerstone for (quantitative) structure activity and structure property predictions—a key field of cheminformatics—is computable properties of graphs. These graphs can be reduced to descriptors or indices based on graph theory, which reflect the physical properties of molecules [2]. Topological indices are numerical values linked with chemical constitution that aim to link chemical structure to physical attributes, chemical reactivity, and biological activity. These distance-based graphical indices are commonly employed to build correlations between molecular graph structure and characteristics. Chemical compounds' physicochemical qualities and bioactivity can be predicted using topological indices [3]. Gao et al. [4] referred chemical and pharmaceutical processes to have advanced rapidly, resulting in the emergence of a slew of novel nanomaterials, crystals, and medications each year. The examination of these various chemicals necessitates a significant number of chemical experiments, which adds to researchers' burden. According to Katritzky et al. [5], their experiments reveal a close link between topological molecule structures and their physical behaviors, chemical properties, and biological traits, such as melting point, boiling temperature, and drug toxicity. Any drug that is effective in the treatment of cancerous disease is known as an anticancer drug, also known as effective anticancer drug. Anticancer medications are divided into various categories, including alkylating agents, antimetabolites, natural compounds, and hormones. Additionally, there are a number of medications that do not fall into those classifications but have anticancer action and are thereby employed in the treatment of cancer. Chemotherapy is sometimes confused with the use of anticancer medications, whereas it refers to the use of chemical compounds to cure cancer in general. Using multicriteria decision-making techniques such as TOPSIS and SAW, this research looked at the behaviors of some drug structures such as anticancer drug SP[*n*]. This is the first research work to rank several drug structures with the help of certain MCDM techniques. TOPSIS is a ranking method that examines decision-making problems quantitatively and qualitatively. It provides the most accurate and timely solutions to our real-world problems than any other MCDM technique. Furthermore, the simplicity, logic, high processing efficiency, and capacity to quantify relative performance for each choice in a simple mathematical form are also advantages of this technique. On the other hand, one of the most basic and widely used weighted average approaches is the simple additive weighting method. This approach has the advantage of being a proportionate linear translation of the original data, which preserves the relative order of the variables. The SAW method demands normalizing the decision matrix to a scale that is comparable to all other ratings currently available.

## 2. Preliminaries

This research paper has considered finite graphs without loops and edges [6]. Let us consider a simple graph *G*(*p*, *q*) with vertex set *V*(*G*) = {*v*_1_′, *v*_2_′, *v*_3_′, ⋯, *v*_*n*_′} and edge set *E*(*G*) with |*V*(*G*)| = *q*, |*E*(*G*)| = *p*. The number of edges connected to vertex *p* ∈ *V*(*G*) is called degree and is denoted by *d*_*G*_(*p*).

In 1975, the topological connectivity index RI(*G*) of a graph *G* defined as the sum of weights was proposed by Randić [7], i.e.,

(1) $$\begin{array}{c} \text{RI}\left(G\right)=\underset{u^{\prime }v^{\prime }\in E\left(G\right)}{\sum }\frac{1}{\sqrt{d_{G}\left(u^{\prime }\right)d_{G}\left(v^{\prime }\right)}}. \end{array}$$

This index was originally known as the “branching index” or “molecular connectivity index,” and it was found to be useful in determining the level of branching. The Randić index is the name given to this parameter nowadays [8, 9]. Bollobás and Erdös [10] expanded this index in 1998 by substituting any real number for −1/2 to produce the general Randić index RI_*α*_. Thus,

(2) $$\begin{array}{c} \text{RI}\left(G\right)=\underset{u^{\prime }v^{\prime }\in E\left(G\right)}{\sum }{\left(d_{G}\left(u^{\prime }\right)d_{G}\left(v^{\prime }\right)\right)}^{\alpha }. \end{array}$$

Randić has demonstrated a link between the Randić index and a variety of physiochemical properties [11, 12]. Recently, Dvořák et al. [13] have shown if we have RI(*G*) ≥ rad(*G*)/2, where rad(*G*) is the radius of *G*. The main point of their work was to introduce a new index, RI′(*G*), which was defined as

(3) $$\begin{array}{c} {\text{RI}}^{\prime }\left(G\right)=\underset{u^{\prime }v^{\prime }\in E\left(G\right)}{\sum }\frac{1}{\max\left\{d_{G}\left(u^{\prime }\right),d_{G}\left(v^{\prime }\right)\right\}}. \end{array}$$

Using this index, Cygan et al. [14] showed that, for any connected graph *G* of maximum degree at most four that is not a path with an even number of vertices, (*G*) ≥ rad(*G*). Consequently, they resolve the conjecture RI(*G*) ≥ rad(*G*) − 1 specified by Zhang et al. [15]. They demonstrated that the inequality holds for all connected chemical graphs *G*, RI′(*G*) ≥ rad(*G*)–1/2 holds.

Furtula et al. [16] recently suggested the enhanced Zagreb index (AZI), a new topological measure based by the ABC index defined as

(4) $$\begin{array}{c} \text{AZI}\left(G\right)=\underset{u^{\prime }v^{\prime }\in E\left(G\right)}{\sum }{\left(\frac{\text{ⅆ}\left(u^{\prime }\right)+\text{ⅆ}\left(v^{\prime }\right)}{\text{ⅆ}\left(u^{\prime }\right)+d\left(v^{\prime }\right)-2}\right)}^{3}, \end{array}$$

whose predictive power exceeds that of the ABC index. He revealed that the AZI is a useful predictor of the heat of formation in heptanes and octanes [17]. It is possible to conclude that only this index passed the tests used in this investigation. As a result, when creating quantitative structure–property relationships, this index should be used [18]. Gao et al. [19] defined the forgotten topological index (or, F-index) which is stated as

(5) $$\begin{array}{c} \text{FI}\left(G\right)=\underset{u^{\prime }v^{\prime }\in E\left(G\right)}{\sum }\left(d{\left(u^{\prime }\right)}^{2}+d{\left(v^{\prime }\right)}^{2}\right). \end{array}$$

De et al. [20] presented some basic properties of the forgotten topological index and demonstrated how this index can improve the Zagreb index's physical-chemical applicability.

## 3. Drug Structures

**Figure 1:**
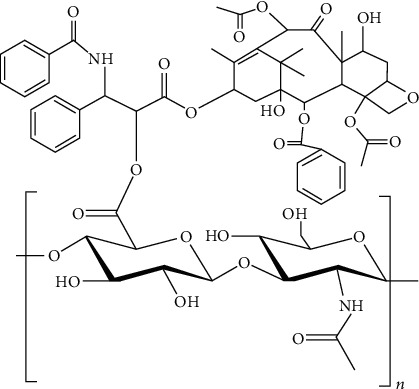
Molecular graph of HA-paclitaxel conjugates.

In this research paper, we consider several molecular structures of drugs along with their physiochemical properties, i.e., molecular weight, melting point, boiling point, complexity, and density. Disaccharide, its basic structure, has a high energy stability [21]. As a fast-developing platform for targeting CD44-overexpressing cells, HA is a promising cancer treatment [22]. HA works well as a drug transporter and a drug target. Increased water solubility and activity preservation are the great attributes of HA-PTX conjugates; more importantly, they could be applied as targeted drug delivery to boost antitumor efficacy [23].  Figure 1 depicts the structure of hyaluronic acid-paclitaxel conjugates.

**Figure 2:**
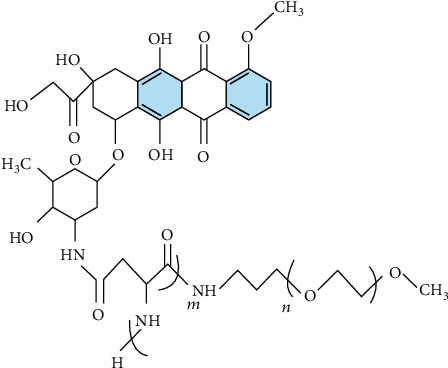
Chemical graph of SP[*n*].

The Dox-loaded micelle containing poly-(ethylene glycol)-poly(aspirate) PEG-PAsp block copolymer with chemically conjugated Dox (SP[n]) is depicted in Figure 2.

According to Nishiyama and Kataoka [24], it is a well-known smart polymer family that is used as an anthracycline anticancer antibiotic and is used to treat a variety of cancers. As a result, it possesses strong anticancer properties and is widely utilized in pharmaceuticals. The integer number *n* is the step of growth in this form of polymer, as seen in Figure 2.

**Figure 3:**
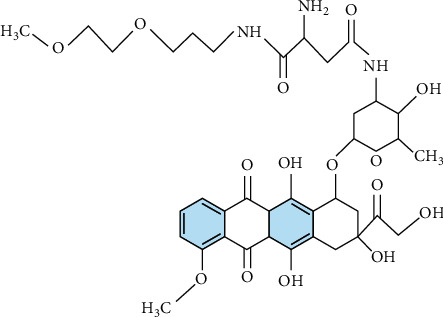
Chemical graph of SP[*n*] for *n* = 1.

**Figure 4:**
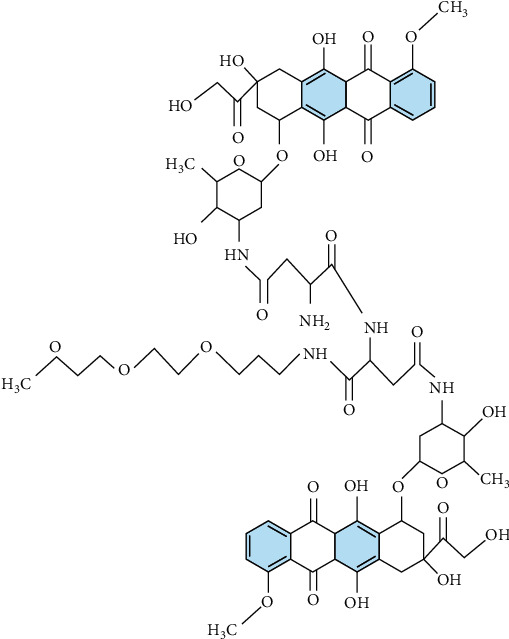
Chemical graph of SP[*n*] for *n* = 2.

**Figure 5:**
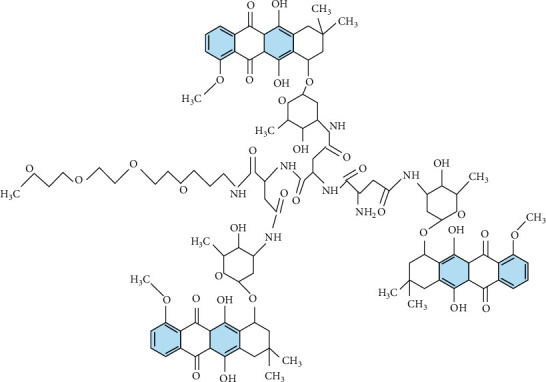
Chemical graph of SP[*n*] for *n* = 3.

When *n* = 1, 2, 3 (see Figures 3–5, respectively).

**Figure 6:**
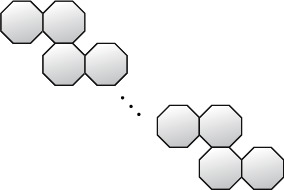
The zig-zag chain of 8-cycle *Z*_*n*_.

A polyomino system is a finite 2-connected plane system in which each internal face (also known as a cell) is enclosed by a one-length regular square [25, 26], which contains applications of polyomino systems to crystal physics. A polyomino chain is a polyomino system with a path as its inner dual graph (see Figure 6). It will be denoted by *Z*_*n*_.

**Figure 7:**
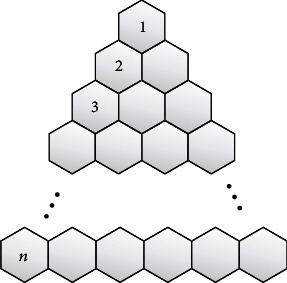
Molecular graph of triangular benzenoid *T*_*n*_.

Now, look at the graph of triangular benzenoids *T*_*n*_, where *n* is the number of hexagonal structures in the base graph.  Figure 7 clearly shows that *T*_*n*_ has 1/2*n*(*n* + 1) hexagons [27]. It is crucial in pharmacy drug design and a variety of other applications.

**Figure 8:**
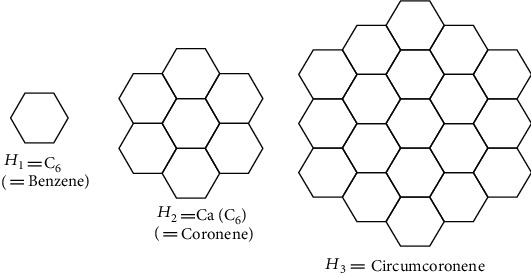
Renowned members of circumcoronene benzenoid series *H*_*k*_ for *k* ≥ 1.

**Figure 9:**
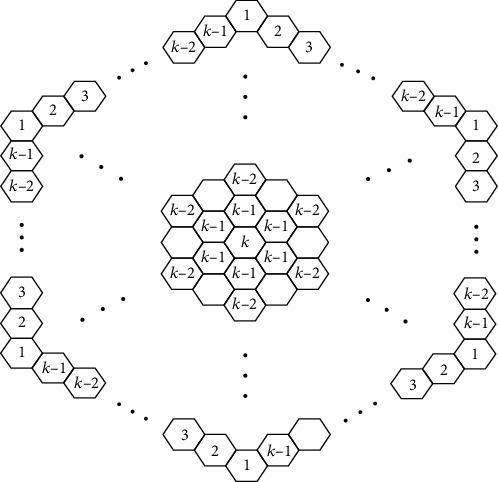
The molecular graph of *H*_*k*_ for *k* ≥ 1.

We derive the circumcoronene series of benzenoid after generalizing benzene molecules [28]. Benzene is significant in chemistry because it aids in the production of aromatic compounds. The benzenoid series circumcoronene consists of several copies of benzene *C*_6_ on the perimeter (Figures 8 and 9). One family of benzenoid *H*_*k*_ that arises from the benzene molecule is the circumcoronene series. Coronene *H*_2_ orCa(*C*_6_), the first term of the Capra-designed planar benzenoid series Ca_*n*_(*C*_6_), is a well-known member of this family (*C*_6_).

## 4. Some Important Results

In this section, we emphasize on calculating the additive degree-based topological indices of the molecular graphs.

- Additive degree-based topological invariants of conjugated Dox SP [*n*]

Let *G* be the graph of Dox-loaded micelle comprising PEG-PAsp block copolymer with chemically conjugated Dox (SP [*n*]). Then, we have

(6) $$\begin{array}{c} R_{1}\left(G\right)=335n+15,\\ R_{-1}\left(G\right)=10.611n+1.333,\\ R_{1/2}\left(G\right)=131.6286n+8.69677,\\ AZ\left(G\right)=444\cdot 8193n+19.375,\\ F\left(G\right)=744n+34. \end{array}$$

From [6], the molecular graph of (SP[*n*]) contains 49*n* + 1 vertices and 54*n* + 5 edges.

(ii) Additive degree-based topological invariants of hyaluronic acid-paclitaxel conjugates *G*_*n*_

Let *G* be graph of hyaluronic acid-paclitaxel conjugates *G*_*n*_ . Then, we have

(7) $$\begin{array}{c} R_{1}\left(G\right)=629n-11,\\ R_{-1}\left(G\right)=19.2278n-0.0278,\\ R_{1/2}\left(G\right)=243.1083n-3.4494,\\ AZ\left(G\right)=822.5972n-11.3906,\\ F\left(G\right)=1404n+23. \end{array}$$

From [21], the molecular graph of (*G*_*n*_) contains 87*n* vertices and 96*n* edges.

(iii) Additive degree-based topological invariants of polyomino chain of *n*-cycle *Z*_*n*_

Let *G* be graph of polyomino chain of *n*-cycle *Z*_*n*_. Then, we have

(8) $$\begin{array}{c} {\text{RI}}_{1}\left(G\right)=168n-2,\\ {\text{RI}}_{-1}\left(G\right)=5.2222n+0.7778,\\ {R\text{I}}_{1/2}\left(G\right)=67.5959n+2,\\ \text{AZI}\left(G\right)=251.125n+9.2187,\\ \text{FI}\left(G\right)=344n-4. \end{array}$$

Some of the topological invariants named as *I* and *V* have taken from [29]; the molecular graph of (*Z*_*n*_) contains 24*n* + 2 vertices and 28*n* + 2 edges.

(iv) Additive degree-based topological invariants of circumcoronene series of benzenoid *H*_*k*_, *k* ≥ 1

Let *G* be graph of circumcoronene series of benzenoid *H*_*k*_, *k* ≥ 1. Then, we have

(9) $$\begin{array}{c} {\text{RI}}_{1}\left(G\right)=81k^{2}-63k+6,\\ {\text{RI}}_{-1}\left(G\right)=k^{2}+0.333k+0.1667,\\ {\text{RI}}_{1/2}\left(G\right)=27k^{2}-15.6061k+0.60612,\\ \text{AZI}\left(G\right)=102.5156k^{2}-74.8593k+20.3437,\\ \text{FI}\left(G\right)=162k^{2}-114k. \end{array}$$

From [29], the molecular graph of *H*_*k*_, *k* ≥ 1 contains 6*k*^2^ + 6*k* − 6 vertices and 9*k*^2^ − 3*k* edges.

(v) Additive degree-based topological indices of triangular benzenoid *T*_*n*_

Let *G* be graph of triangular benzenoid *T*_*n*_. Then, we have

(10) $$\begin{array}{c} {\text{RI}}_{1}\left(G\right)=9n^{2}+45n-30,\\ {\text{RI}}_{-1}\left(G\right)=0.25n^{2}+0.4167n+0.8333,\\ {\text{RI}}_{1/2}\left(G\right)=3.6742n^{2}-14.3257n-6,\\ \text{AZI}\left(G\right)=12n^{2}+56.3437n-20.3437,\\ \text{FI}\left(G\right)=19.5n^{2}+88.5n-60. \end{array}$$

From [29], the molecular graph of *T*_*n*_ contains *n*^2^ + 4*n* + 1 vertices and ((3/2)3/2*n*^2^) + (9/2) edges.

The objectives of this paper are to give behavioral analysis of chemical structures of anticancer drug molecules using several topological indices, such as the Randić index and the augmented Zagreb index, as well as the forgotten topological index. We will also present a weighted evaluation of several topological indices in this research endeavor, as chemical invariants aim to provide a less expensive and more efficient means for scientists and analysts to determine the physical and chemical features of anticancer medications. Two different decision-making techniques will be used to carry out this weighted evaluation. The Approach for Order Preference by Similarity to Ideal Solution (TOPSIS) will be the first technique. This weighted evaluation will be carried out for the ideal solution and the greatest distance from the worst solution. It also tries to use mathematics to assess the accuracy of molecular compound specifications. This method of multicriteria decision-making first appeared in the 1980s (MCDM).

**Figure 10:**
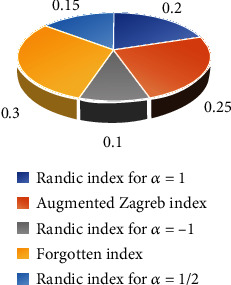
Allocation of weights

(i) Allocation of weights: weights show how much of a drug structure should be taken into account. It is beneficial to have a drug structure with a wide range of physical and chemical properties. In that situation, we give them a lot more weight in comparison to the others and the others do as well (see Figure 10). The weight is allocated according the formula mentioned below
  (11) $$\begin{array}{c} \overset{j}{\underset{i=1}{\sum }}W_{j}^{\prime }=1. \end{array}$$
(ii) A drug's impact refers to whether it has a positive or negative impact. For example, which physiochemical feature is ideal best and which is ideal worst for our drug structure. The data values for a certain factor should be regarded as standard units
(iii) Ideal best and ideal worst: we must first deal with the properties of our concerned drug structures and then correlate the abovementioned attributes with physical properties of every drug in order to determine the ideal best and ideal worst. The molecular weight, density, complexity, boiling point, and melting point are five common properties of drug structures. The solid density of pharmacological substances, from powder to tablet, is an important feature. It enables us to determine which chemicals will sink in a liquid. If the density of the substance is less than the density of the liquid in which it is immersed, it will flow [30]. As a result, low density is optimal for our pharmacological structures. The melting point is a fundamental physical feature that defines the transition in pharmaceutical sciences, chemical, and biological chemistry. In general, melting points with lower melting points are more likely to be absorbed than melting points with higher melting points. Another key attribute employed in the pharmaceutical industry is molecular weight. The degree of crystallinity of the polymer increased as the molecular weight of the polymer decreased [31]. The drug structures have a molecular weight of less than 1000 g/mol; hence, we use low molecular weight pharmaceuticals. The boiling point of a medicine is one of its most important characteristics [32]. It is for storing and carrying things. We have more storage for our pharmaceuticals if the boiling point is higher. Drug treatment complexity is acknowledged to be a risk factor for administration errors and nonadherence, resulting in increased healthcare expenses [33]

### 4.1. TOPSIS

Assume that each property is evaluated independently. Comparing the measure of similarity to the ideal alternative could be used to rate compromises [34]. From Table 1, there are *m* alternatives (drug structures) and *n* attributes (Randić indices, augmented Zagreb index, and forgotten topological index). In this regard, we attempt to set appropriate weights for the attributes in order to make the best decision and strike a balance between them [35].

**Step 1 .**

Selecting the important attributes and constituting the decision matrix based on *m* alternatives (drug structures) and *n* attributes (Randić indices, augmented Zagreb Index, and forgotten topological index) in Table 2:

(12) $$\begin{array}{c} D_{ij}=\left[\begin{array}{cccc} d_{11} & d_{12} & \cdots & d_{1n}\\ d_{21} & d_{22} & \cdots & d_{2n}\\ \vdots & \vdots & \vdots & \vdots \\ d_{n1} & d_{n2} & \cdots & d_{mn} \end{array}\right]. \end{array}$$

Now, we construct our decision matrix *D*_*ij*_, that is

**Step 2 .**

Calculate the normalized decision matrix *H*_*ij*_ (Table 3). The normalized value *r*_*ij*_ of the *i*th alternate (drug structure) with respect to the *j*th attribute (topological indices).

(13) $$\begin{array}{c} H_{ij}=\left[\begin{array}{cccc} h_{11} & h_{12} & \cdots & h_{1n}\\ h_{21} & h_{22} & \cdots & h_{2n}\\ \vdots & \vdots & \vdots & \vdots \\ h_{n1} & h_{n2} & \cdots & h_{mn} \end{array}\right], \end{array}$$

where $H_{ij}=d_{ij}/\sqrt{\sum_{i=1}^{m}d_{ij}^{2}}$∀*j* = 1, 2, 3, ⋯, *n* and *i* = 1, 2, 3, ⋯, *m*.

**Step 3 .**

Calculate the weighted normalized decision matrix *X*_*ij*_ shown in Table 4.

The weighted normalized value is *X*_*ij*_ = *W*_*j*_′.*h*_*ij*_∀*j* = 1, 2, 3, ⋯, *n*, where

(14) $$\begin{array}{c} \overset{j}{\underset{i=1}{\sum }}W_{j}^{\prime }=1. \end{array}$$

Here, we allocate the highest-ranking topological descriptor highest weight. RI_−1_(*G*) gives small values of their respective drug structures so that we assign lowest weight (0.10). Similarly, RI_1/2_(*G*) has slightly different values from RI_−1_(*G*) so we allocate it with little more weight (0.15). Next, if we notice RI_1_(*G*), the values for it are greater than RI_1/2_(*G*) so we assign weight (0.20). Lastly, if we see FI(*G*), that is richest in their values, we give a maximum weight (0.30) to it.

(15) $$\begin{array}{c} W_{j}^{\prime }=0.20,0.25,0.10,0.30,0.15. \end{array}$$

We can calculate the normalized decision matrix using the formula given below.

(16) $$\begin{array}{c} X_{ij}=\left[\begin{array}{cccc} W_{1}^{\prime }h_{11} & W_{1}^{\prime }h_{11} & \cdots & W_{n}^{\prime }h_{1n}\\ W_{1}^{\prime }h_{21} & W_{2}^{\prime }h_{22} & \cdots & W_{n}^{\prime }h_{2n}\\ \vdots & \vdots & \vdots & \vdots \\ W_{1}^{\prime }h_{n1} & W_{2}^{\prime }h_{n2} & \cdots & W_{n}^{\prime }h_{mn} \end{array}\right]. \end{array}$$

**Step 4 .**

Determine the positive ideal solution *L*^+^ and negative ideal solution *L*^−^ (Table 5).

To determine the distance between alternative *i* and the ideal alternative that is defined as

(17) $$\begin{array}{c} L^{+}=\left\{x_{i}^{+},\cdots ,x_{j}^{+}\right\}=\left(\max\left(\text{or }\min\right)\;X_{ij}\left|j\in J\right.\right), \end{array}$$

and distance between alternative *i* and the minimum alternative that is defined as

(18) $$\begin{array}{c} L^{-}=\left\{x_{i}^{-},\cdots ,x_{j}^{-}\right\}=\left(\min\left(\text{or }\max\right)\;X_{ij}\left|j\in J\right.\right). \end{array}$$

**Step 5 .**

Compute the separation measure, using the *n*-dimensional Euclidean distance in Table 6. The separation of each alternative form the ideal solution is given by

(19) $$\begin{array}{c} P_{i}^{+}=\sqrt{\overset{n}{\underset{j=1}{\sum }}{\left(X_{ij}-L_{j}^{+}\right)}^{2},}\\ P_{i}^{-}=\sqrt{\overset{n}{\underset{j=1}{\sum }}{\left(X_{ij}-L_{j}^{-}\right)}^{2}.} \end{array}$$

**Step 6 .**

Compute the relative closeness to the ideal solution (Table 7). The relative closeness of *A*_*i*_ with respect to *A* is defined as

(20) $$\begin{array}{c} O_{i}^{*}=\frac{P_{i}^{-}}{P_{i}^{+}+P_{i}^{-}}, \end{array}$$

where 0 < *O*_*i*_^∗^ < 1, *i* = 1, 2, 3, ⋯, *n*.

It is clear that *O*_*i*_^∗^ = 1 if *L*_*i*_ = *L*^+^ and *O*_*i*_^∗^ = 0 if *L*_*i*_ = *L*^−^.

Therefore, a preferable option is the one that poses the value closer to 1.

**Step 7 .**

Rank the reference order based on the descending order of *O*_*i*_^∗^ in Table 8.

### 4.2. SAW

A multicriteria decision-making (MCDM) or multicriteria decision analysis method is the simple additive weighting method (SAW), which is also known as weighted linear combination or scoring method [36]. This method is comprised on the weighted average. The weighted sum of the performance evaluations for every alternative among all attributes is determined using the SAW method [37]. There are different *m* alternatives (drug structures) and *n* attributes (Randić indices, augmented Zagreb index, and forgotten topological index).

The SAW method's compromise ranking algorithm consists of the following steps:

**Step 8 .**

Constitute the decision matrix of *m* alternatives and *n* attributes in Table 9.

(21) $$\begin{array}{c} G_{ij}=\left[\begin{array}{cccc} g_{11} & g_{12} & \cdots & g_{1n}\\ g_{21} & g_{22} & \cdots & g_{2n}\\ \vdots & \vdots & \vdots & \vdots \\ g_{n1} & g_{n2} & \cdots & g_{mn} \end{array}\right]. \end{array}$$

And determine the best *g*_*j*_^+^ and worst *g*_*j*_^−^ values of all the attributes *j* = 1, 2, 3, ⋯, *n*.

**Step 9 .**

By using the abovementioned weighted criteria, we calculate the weights. Also, construct a normalized decision matrix *H*_*ij*_ according to the formula given below, where *m* is the alternatives and *n* is the attributes in Table 10.

(22) $$\begin{array}{c} h_{ij}=\frac{g_{ij}}{\max\left(g_{ij}\right)},\\ h_{ij}=\frac{\min\left(g_{ij}\right)}{g_{ij}}, \end{array}$$

where *i* = 1, 2, 3, ⋯, *m* and *j* = 1, 2, 3, ⋯, *n*.

**Step 10 .**

Evaluate each alternative *M*_*i*_ by the following formula (Table 11):

(23) $$\begin{array}{c} M_{i}=\overset{n}{\underset{j=1}{\sum }}W_{j}h_{ij}, \end{array}$$

where *h*_*ij*_ is the score of *i*th alternative with respect to the *j*th attribute and *W*_*j*_ is the weighted criteria of the attributes.

## 5. Graphical Interpretation of Drug Structures

**Figure 11:**
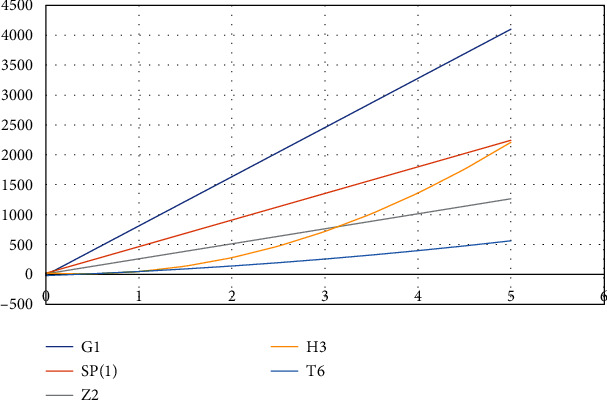
Comparison of alternatives using AZI(*G*).

**Figure 12:**
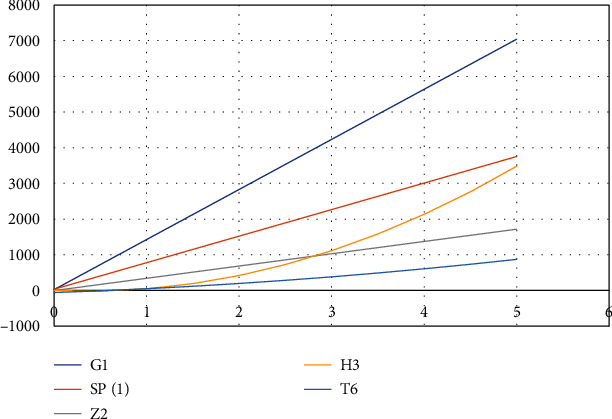
Comparison of alternatives using FI(*G*).

**Figure 13:**
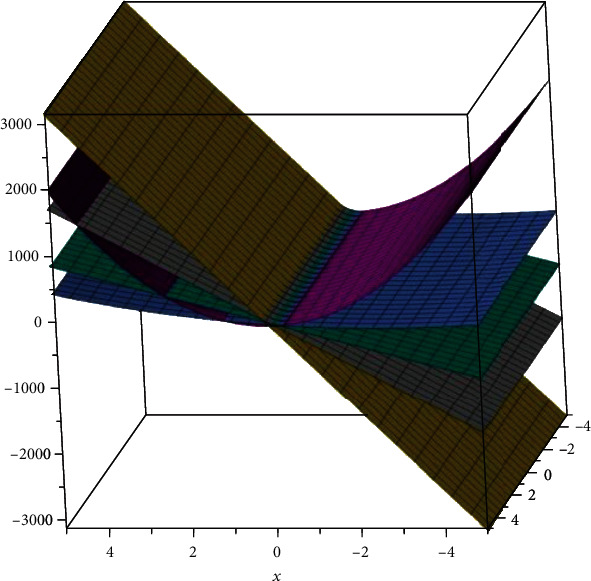
Comparison of alternatives using RI_1_(*G*).

**Figure 14:**
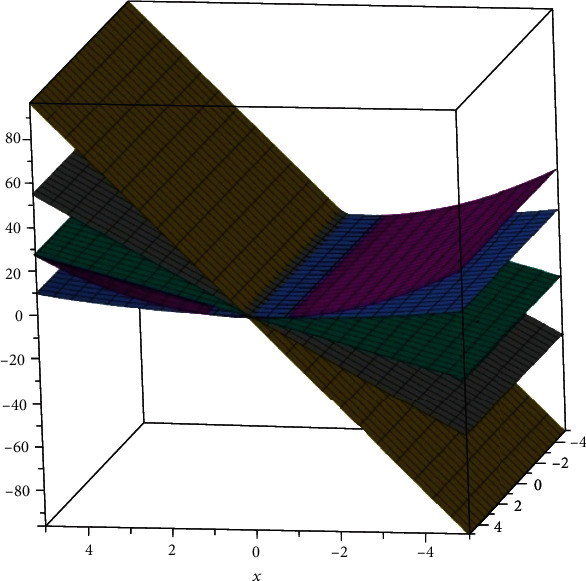
Comparison of alternatives using RI_−1_(*G*).

**Figure 15:**
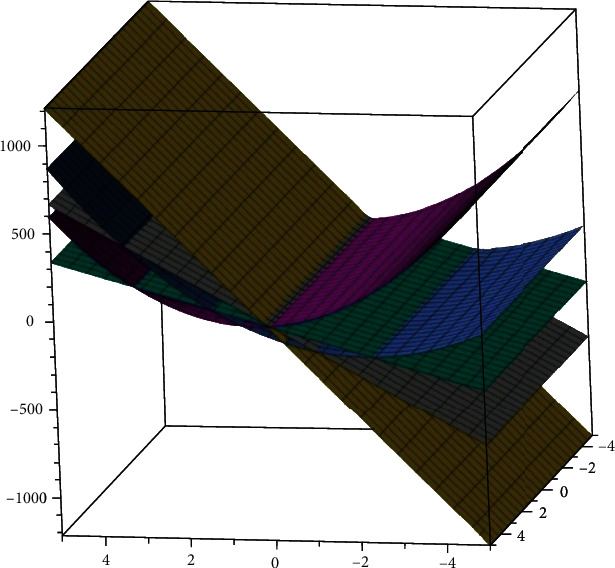
Comparison of alternatives using RI_1/2_(*G*).

The two-dimensional and three-dimensional graphical comparisons of the above results are depicted in Figures 11–15, respectively.

### 5.1. Two-Dimensional Graphs

In both the 2D plots of the drug structures along with the attributes, we have found that *G*_1_ gives us the highest value and *T*_6_ shows the smallest value.

### 5.2. Three-Dimensional Graphs

These 3D graphs are representing the behavior of the drug structures with attributes RI_1_(*G*), RI_−1_(*G*), and RI_1/2_(*G*), respectively. Golden color is indicating *G*_1_ drug structure, grey is indicating SP[1], green is indicating *Z*_2_, Niagara azure is indicating *H*_3_, and purple is indicating *T*_6_ drug structures. In all the graphs, we have clearly seen that *G*_1_ gives us effective role as a drug structure in these plots.

## 6. Conclusion

**Figure 16:**
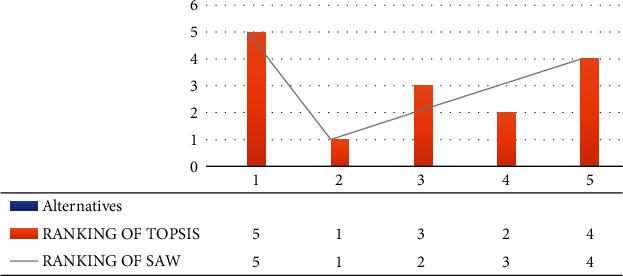
Ranking of TOPSIS and SAW.

Many drug studies reveal strong inner links between the medications' biological and pharmacological properties and their molecular structures. In this research article, using TOPSIS method, *SP*[1] is determined to be the most suitable drug structure as it has close distance to the ideal solution. The drug structures are thus ranked as H_3_, Z_2_, T_6_,and lastly *G*_1_, i.e., *H*_3_ > *Z*_2_ > *T*_6_ > *G*_1_. On the other hand, using SAW, we have observed a slightly changed behavior of drugs as *Z*_2_ and *H*_3_ are ranked opposite in their behaviors. In the SAW method, SP[1] is determined to be the highest ranked drug structure. Other structures are ranked as *Z*_2_ > *H*_3_ > *T*_6_ > *G*_1_. Moreover, the results are plotted using the MS Excel and MAPLE in Figures 11–15, respectively. These theoretical results might be supportive to comprehend the topology of the aforementioned chemical drug structures. The histogram of the ranking is created through the MS Excel as shown in Figure 16. These theoretical results might be helpful to rank the drug structures via chemical invariants in the field of medicine, chemistry, drug discovery, and mathematical chemistry while evaluating these drugs in future.

## Figures and Tables

**Table 1:** Attributes and alternatives.

Alternative	Randić index (*α* = 1)	Augmented Zagreb index	Randić index (*α* = −1)	Forgotten topological index	Randić index (*α* = 1/2)
Hyaluronic acid-paclitaxel conjugates *G*_1_	618	811.2026	19.25	1381	239.6589
Anticancer drug SP[1]	350	464.1943	11.94	778	140.3254
Polyomino chain of *n*-cycle *Z*_2_	334	511.4687	11.22	684	137.1918
Circumcoronene benzenoid series *H*_3_	546	749.7187	10.17	1173	196.7877
Triangular benzenoid *T*_6_	564	718.4062	12.33	1116	212.2270

**Table 2:** Decision matrix *D*_*ij*_.

Alternatives	RI_1_(*G*)	AZI(*G*)	RI_−1_(*G*)	FI(*G*)	RI_1/2_(*G*)
*G*_1_	618	811.2026	19.25	1381	239.6589
SP[1]	350	464.1943	11.94	778	140.3254
*Z*_2_	334	511.4687	11.22	684	137.1918
*H*_3_	546	749.7187	10.17	1173	196.7877
*T*_6_	564	718.4062	12.33	1116	212.2270

**Table 3:** Normalized decision matrix *H*_*ij*_.

Alternatives	RI_1_(*G*)	AZI(*G*)	RI_−1_(*G*)	FI(*G*)	RI_1/2_(*G*)
*G*_1_	0.2562	0.2492	0.2965	0.2568	0.2587
SP[1]	0.1451	0.1426	0.1839	0.1515	0.1515
*Z*_2_	0.1284	0.1571	0.1728	0.1332	0.1481
*H*_3_	0.2263	0.2303	0.1566	0.2285	0.2124
*T*_6_	0.2338	0.2208	0.1899	0.2174	0.2291

**Table 4:** Weighted normalized decision matrix *X*_*ij*_.

Alternatives	RI_1_(*G*)	AZI(*G*)	RI_−1_(*G*)	FI(*G*)	RI_1/2_(*G*)
Weight *W*_*j*_	**0.20**	**0.25**	**0.10**	**0.30**	**0.15**
*G*_1_	0.0512	0.0623	0.0296	0.0770	0.0388
SP[1]	0.0290	0.0356	0.0184	0.0454	0.0227
*Z*_2_	0.0276	0.0393	0.0172	0.0399	0.0222
*H*_3_	0.0452	0.0575	0.0156	0.0628	0.0318
*T*_6_	0.0467	0.0552	0.0189	0.0652	0.0343

**Table 5:** Calculation of the positive ideal solution *L*^+^ and negative ideal solution *L*^−^.

Alternatives	RI_1_(*G*)	AZI(*G*)	RI_−1_(*G*)	FI(*G*)	RI_1/2_(*G*)
Properties	Molecular weight	Complexities	Density	Boiling point	Melting point
Weight *W*_*j*_	0.20	0.25	0.10	0.30	0.15
*G*_1_	0.0512	0.0623	0.0296	0.0770	0.0388
SP[1]	0.0290	0.0356	0.0184	0.0454	0.0227
*Z*_2_	0.0276	0.0393	0.0172	0.0399	0.0222
*H*_3_	0.0452	0.0575	0.0156	0.0628	0.0318
*T*_6_	0.0467	0.0552	0.0189	0.0652	0.0343
*L* ^+^(ideal best)	0.0276	0.0356	0.0156	0.0770	0.0222
*L* ^−^(ideal worst)	0.0512	0.0623	0.0296	0.0399	0.0388

**Table 6:** Calculate the separation measures *P*_*i*_^+^ and *P*_*i*_^−^.

Alternatives	RI_1_(*G*)	AZI(*G*)	RI_−1_(*G*)	FI(*G*)	RI_1/2_(*G*)	*P*_*i*_^+^	*P*_*i*_^−^
*G*_1_	0.0512	0.0623	0.0296	0.0770	0.0388	0.0416	0.0370
SP[1]	0.0290	0.0356	0.0184	0.0454	0.0227	0.0317	0.0402
*Z*_2_	0.0276	0.0393	0.0172	0.0399	0.0222	0.0372	0.0369
*H*_3_	0.0452	0.0575	0.0156	0.0628	0.0318	0.0309	0.0335
*T*_6_	0.0467	0.0552	0.0189	0.0652	0.0343	0.0323	0.0290

**Table 7:** Computation of relative closeness to the ideal solution *O*_*i*_^∗^.

*P*_*i*_^+^	*P*_*i*_^−^	*O*_*i*_^∗^
0.0416	0.0370	0.4706
0.0317	0.0402	0.5592
0.0372	0.0369	0.4975
0.0309	0.0335	0.5206
0.0323	0.0290	0.4730

**Table 8:** Rank the alternatives.

Alternatives	*O*_*i*_^∗^	Rank
*G*_1_	0.4706	5
SP[1]	0.5592	1
*Z*_2_	0.4975	3
*H*_3_	0.5206	2
*T*_6_	0.4730	4

**Table 9:** The decision matrix *G*_*ij*_.

Alternatives	*R*_1_(*G*)	AZ(*G*)	*R*_−1_(*G*)	*F*(*G*)	*R*_1/2_(*G*)
*G*_1_	618	811.2026	19.25	1381	239.6589
SP[1]	350	464.1943	11.94	778	140.3254
*Z*_2_	334	511.4687	11.22	684	137.1918
*H*_3_	546	749.7187	10.17	1173	196.7877
*T*_6_	564	718.4062	12.33	1116	212.2270
Best (*g*_*j*_^+^)	334	464.1943	10.17	1381	137.1918
Worst (*g*_*j*_^−^)	618	811.2026	19.25	684	239.6589

**Table 10:** Normalized decision matrix *H*_*ij*_.

Alternatives	*R*_1_(*G*)	AZ(*G*)	*R*_−1_(*G*)	*F*(*G*)	*R*_1/2_(*G*)
Weight *W*_*j*_	**0.20**	**0.25**	**0.10**	**0.30**	**0.15**
*G*_1_	0.540453	0.57223	0.528312	1	0.572446
SP[1]	0.954286	1	0.851759	0.56336	0.977669
*Z*_2_	1	0.907571	0.906417	0.495293	1
*H*_3_	0.611722	0.619158	1	0.849385	0.697156
*T*_6_	0.592199	0.646145	0.824818	0.80811	0.646439

**Table 11:** Rank the alternatives.

Alternatives	*M*_*i*_	Rank
*G*_1_	0.689846	5
SP[1]	0.841691	1
*Z*_2_	0.816123	2
*H*_3_	0.736523	3
*T*_6_	0.701856	4

## Data Availability

The data used to support this work are cited within the text as references.
